# The Story of the Insane from Year to Year

**Published:** 1899-11-18

**Authors:** 


### THE STORY OF THE INSANE FROM YEAR TO YEAR.

Twelfth Series.

### OXFORD COUNTY ASYLUM AT LITTLEMORE.

There is, apparently, a mistake in the date attached to Table I., but it would seem that the asylum contained 533 patients on December 31st, 1898; this number, however, included two absent on trial. There were 93 first admissions, 18 readmissions, 41 recoveries, 10 discharged relieved, and 58 deaths. The recoveries stand at 21 per cent. on the admissions, and the deaths at 11 per cent. on the average number resident; the former being very low, and the latter rather high. Nineteen of the deaths were caused by cerebral and spinal diseases, and 17 by lung diseases. Of the year's admissions, 10 are supposed to have been driven insane by such moral causes as domestic trouble and mental anxiety, 6 by hereditary tendency, and 16 by intemperance in drink. There were three cases of measles and three of enteric fever, and of the latter one died. The visitors have not decided to enlarge the asylum, although it has been practically full during the year, and they point out that there are 156 lunatics in the workhouses or with their relations, and that some of these might be sent to the asylum at any time. Instead of pushing on with the additions, they suggest that as a temporary expedient patients be transferred under contract to another asylum. We believe this is leaning on a broken reed. It is quite possible that no asylum could be found within reasonable distance to take the patients in, and if one could be found, the Oxford Committee would have to pay 14s. a week for every patient removed. A simple calculation will show what a pecuniary loss the Oxford ratepayer would suffer, and the question would only be shelved for a time. The want of additional land need not deter from action as fifty acres of land are plenty for 750 patients. The charge to the unions for maintenance was a little over 7s. a week per head.

### THE LAWN ASYLUM, LINCOLN.

This is one of the lunatic hospitals managed by a committee which we have in former years noticed favourably, and we are pleased to be able to state again that it has continued its usefulness during 1898. There were 30 first admissions, 5 readmissions, 12 recoveries, 10 discharged relieved, and 6 died. At the close of the year the hospital contained 81 patients, and 112 were under treatment during the year. In addition to these there were several voluntary boarders. The committee say they wish to "emphasise their contention that in no institution in the kingdom where the mentally afflicted are provided for can better surroundings be secured," and they add that the "percentages of recoveries and reliefs testify to the advantages secured by a term of residence at this institution." The committee are, however, aware that improvements and enlargements ought to be carried out soon; but they very wisely delay the work until they have sufficient funds in hand. Something may be done by slightly raising the charges for those patients who can afford to pay, but on the other hand it is most desirable that no increase should be made to the rates for poorer patients. Surely donations might be forthcoming if the charitably disposed among the public were aware of the benefits which would flow from their gifts. The greater portion of the patients are ladies, and we are glad to notice that the assistant medical officer is a lady.

### THE DERBY BOROUGH ASYLUM AT ROWDITCH.

On January 1st, 1898, this asylum contained 318 patients, and by the end of December the number had risen to 324. There were 72 first admissions, 10 readmissions, 32 recoveries, and 25 deaths. Calculated on the admissions, the recoveries reach the respectable percentage of 42·6, and the deaths, calculated on the average number resident, are only 7·8 per cent. Cerebral and spinal diseases account for seven of the deaths, consumption for four, and pleurisy for one. Moral causes account for the insanity in 23 of the admissions, intemperance in drink for 22, and hereditary influence for 25; thus demonstrating clearly enough that over 50 per cent. of the insanity in the borough of Derby ought never to have existed at all, and never would have existed had the laws of morality and common-sense been attended to. It is painful to have to notice similar statistics in the reports of every asylum throughout this Christian England of ours, and it is still more painful to know that so little is being done to stem this torrent of madness. There is generally something interesting in Dr. Macphail's reports, and this year he gives an able summary of the first ten years of the life of the asylum. We notice that the ratio of first admissions per 10,000 of the population of Derby was 5·15 in 1889, and that it was 5·91 in 1898, but it has been as high as 6·79 and as low as 4·55 in other years. These statistics naturally lead to the consideration of the much-discussed question of the increase in the numbers of the insane, and Dr. Macphail is of opinion that there is no real increase of insanity, but that there is an increase in the number of insane persons kept in asylums; and we must admit that Dr. Macphail makes good his position as far as the borough of Derby is concerned. He attributes the increase in asylum lunatics to several causes, and among others to the proximity of the asylum to the centre from which it draws its population, to a reduction in the numbers of patients treated in workhouses, and to the increased longevity of asylum patients.

The visitors say that "Dr. Macphail continues to give the committee the amplest grounds for confidence in his economical and efficient management of the asylum, and in his care and treatment of the patients." We notice that nine holders of the medico-psychological certificate resigned during the year, five of them to engage in private nursing. It is just possible that the training received and the certificate obtained may slightly increase the changes among the nursing staff, but it cannot have much effect. We should much like to hear that Dr. Macphail had commenced a thorough system of three years' training and annual examinations. The weekly charge to the union was 10s. 4d. a head.

